# High Prevalence of Metabolic Syndrome Features in Patients Previously Treated for Nonfunctioning Pituitary Macroadenoma

**DOI:** 10.1371/journal.pone.0090602

**Published:** 2014-03-07

**Authors:** Sjoerd D. Joustra, Kim M. J. A. Claessen, Olaf M. Dekkers, André P. van Beek, Bruce H. R. Wolffenbuttel, Alberto M. Pereira, Nienke R. Biermasz

**Affiliations:** 1 Center for Endocrine Tumors Leiden, Department of Endocrinology and Metabolism, Leiden University Medical Center, Leiden, Netherlands; 2 Department of Clinical Epidemiology, Leiden University Medical Center, Leiden, Netherlands; 3 Department of Endocrinology, University of Groningen, University Medical Center Groningen, Groningen, Netherlands; University of Leicester, United Kingdom

## Abstract

**Objective:**

Patients treated for nonfunctioning pituitary macroadenoma (NFMA) with suprasellar extension show disturbed sleep characteristics, possibly related to hypothalamic dysfunction. In addition to hypopituitarism, both structural hypothalamic damage and sleep restriction per se are associated with the metabolic syndrome. However, the prevalence of the metabolic syndrome in patients with NFMA is not well established. Our objective was to study the prevalence and risk factors for (components of) the metabolic syndrome in patients treated for NFMA.

**Design:**

The metabolic syndrome (NCEP-ATP III criteria) was studied in an unselected cohort of 145 NFMA patients (aged 26–88yr, 44% female) in long-term remission after treatment, receiving adequate stable hormone replacement for any pituitary deficiencies. The results were compared to population data of 63,995 Dutch inhabitants by standardization (*LifeLines* cohort study).

**Results:**

NFMA patients showed increased risk for reduced HDL-cholesterol (SMR 1.59, 95% CI 1.13–2.11), increased triglyceride levels (SMR 2.31, 95% CI 1.78–2.90) and the metabolic syndrome (SMR 1.60, 95% CI 1.22–2.02), but not for increased blood pressure, waist circumference or hyperglycemia. Preoperative visual field defects independently affected the risk for increased blood pressure (OR 6.5, 95% CI 1.9–22.2), and hypopituitarism was associated with a body mass index - dependent risk for increased waist circumference (OR 1.6, 95% CI 1.2–2.2) and the metabolic syndrome (OR 1.4, 95% CI 1.0–1.9).

**Conclusions:**

Patients treated for NFMA are increased at risk for developing the metabolic syndrome, mainly due to decreased HDL-cholesterol and increased triglycerides. Risk factors included hypopituitarism and preoperative visual field defects. Hypothalamic dysfunction may explain the metabolic abnormalities, in addition to intrinsic imperfections of hormone replacement therapy. Additional research is required to explore the relation between derangements in circadian rhythmicity and metabolic syndrome in these patients.

## Introduction

Nonfunctioning pituitary macroadenomas (NFMA, adenoma >10 mm) comprise approximately 25% of clinically relevant pituitary adenomas [1], and often present with pituitary insufficiency and visual field defects (VFD) due to compression of the optic chiasm. Transsphenoidal surgery is the treatment of choice, resulting in improvement of visual function in the majority of patients [2]. Selected patients require treatment by postoperative radiotherapy [3]. In follow-up, a high prevalence of hypopituitarism remains present in NFMA patients.

Previously, we have shown that patients successfully treated for NFMA suffer from disturbances in sleep characteristics, circadian movement rhythm, and subjective sleep quality [4], the cause of which remains to be elucidated. In view of the close anatomical relationship between the optic chiasm and the hypothalamus, it is possible that impaired function of the suprachiasmatic nucleus (SCN) of the hypothalamus, the main director of circadian rhythmicity, might cause these symptoms.

Besides the decreased quality of life that was associated with these sleeping difficulties [4], the clinical relevance and consequences of possible circadian dysregulation in NFMA patients might be further underlined and assessed by an increased risk for cardiovascular disease. Circadian dysregulation due to hypothalamic damage is associated with the metabolic syndrome both directly, a syndrome known as hypothalamic obesity after structural hypothalamic damage [5], and indirectly, through the consequences of decreased sleep duration and quality [6]. In addition, intrinsic imperfections of hormone replacement therapy might increase the risk for the metabolic syndrome in NFMA patients.

To date, most studies on metabolic features in patients with pituitary diseases were performed in heterogeneous patient groups with either hormone excess syndromes or GH deficiency (GHD), which makes it cumbersome to explore potential hypothalamic effects of pituitary tumors. Therefore, in the present study, we studied the metabolic syndrome and its individual components in an unselected cohort of patients treated for NFMA, and compared these to data from a large Dutch population study by standardization.

## Patients and Methods

### Ethics statement

The Medical Ethics Committee of the Leiden University Medical Center issued a statement of no objection. Patients gave written consent for the collection of data from outside our hospital.

### Study design

In this controlled cross-sectional study, the prevalence of the metabolic syndrome and its components were studied in an unselected cohort of 145 patients treated for NFMA, and compared to population based normative data of 63,995 Dutch inhabitants by standardization (*LifeLines* cohort study).

### Subjects

#### NFMA Patients

Based on a clinical database of 226 patients consecutively treated for NFMA at our center between 1980 and 2010, data and parameters of the metabolic syndrome were collected from clinical records. Patients were included when a) treated for NFMA via transsphenoidal surgery, b) any anterior pituitary hormone deficiencies were adequately and stably substituted (*vide infra*), and c) data on all parameters of the metabolic syndrome was retrievable. 81 patients (36%) were excluded due to missing data. The excluded patients did not differ statistically significant from the included patients, except for fewer cases of radiotherapy and generally a higher age in excluded patients.

The NFMA was staged according to Wilson’s modification of the classification by Hardy [7]. VFD were diagnosed by standard Goldmann perimetry and evaluated by an ophthalmologist. Patients were routinely evaluated every year, and data from the most recent visit were used. Eleven patients were followed yearly in other hospitals, and their data were obtained from treating physicians. In 10 deceased patients, data from their last visit were used.

ACTH deficiency was defined as an insufficient increase in cortisol levels (<0.55 μmol/liter) after a CRH stimulation test or insulin tolerance test. TSH deficiency was defined as free T4 levels below the reference range (<10 pmol/L). Hypogonadism was defined as low testosterone levels (<8.0 nmol/liter) in men, and absence of menstrual cycle for more than one year in the presence of low estrogen levels in premenopausal women. GHD was defined as a GH peak response to the insulin tolerance test (ITT) below 3μg/liter (glucose nadir <2.2 mmol/liter) or GH releasing hormone - arginine test [with body mass index (BMI) – adjusted GH cutoffs] in case of contraindications for ITT, according to guidelines [8]. Data from the most recent hormonal axis evaluation were used. Hypopituitarism was supplemented by hydrocortisone, levothyroxine, recombinant human GH (rhGH, unless contraindicated or not preferred), testosterone in men, and estrogen in combination with prostagens in premenopausal women. Dosages were monitored and adjusted as required, and stable substitution was assumed if medication was not adjusted for 6 months, complaints were absent, and basal hormone levels were normal. Lipid-lowering, antihypertensive and antidiabetic drugs were started at the discretion of the treating physicians.

#### General population

Population data were derived from the *Lifelines study* [9], a cohort follow-up study in a random sample of 165,000 inhabitants of the northern part of the Netherlands covering three generations. All participants provided written informed consent before participating in the study. The medical ethical review committee of the University Medical Center Groningen approved the study protocol.

For the present cross-sectional study we included subjects of Western European origin and age of 18–80 years who participated between December 2006 and January 2012. The total number of individuals included was 63,995.

### Criteria for the metabolic syndrome

The criteria for the metabolic syndrome as imposed by the National Cholesterol Education Program Adult Treatment Panel III, last updated in 2005 by the American Heart Association [10], were used. These criteria have been extensively evaluated [11], [12], the majority of reports being supportive of the criteria posing a useful and accurate prediction of cardiovascular risk. Metabolic syndrome was defined as having any three of the following:

- Waist circumference ≥102 cm in men and ≥88 cm in women. If waist circumference was unknown, patients with a BMI of over 30.0 kg/m^2^ were assumed to have an elevated waist circumference. This threshold serves as a conservative indication of elevated waist circumference [13], and is used in the criteria of the International Diabetes Foundation [14].
- Triglycerides ≥150 mg/dL, or specific drug treatment.
- HDL-cholesterol <40 mg/dL in men and <50 mg/dL in women, or specific drug treatment.
- Blood pressure ≥130 mmHg systolic or ≥85 mmHg diastolic, or antihypertensive treatment.
- Fasting glucose ≥100 mg/dL, or antidiabetic treatment.

The criteria state that fibrates and nicotinic acid are the most commonly used drugs for elevated triglycerides and reduced HDL-cholesterol, implying that statins are not considered specific treatment for those conditions. Indeed, statins predominantly lower LDL-cholesterol and have only a minor indirect effect on triglyceride and HDL-cholesterol levels [15], [16]. Therefore, in this study only fibrates and nicotinic acid were considered specific treatment for elevated triglycerides or reduced HDL-cholesterol.

### Assays

From 1986 to 2005, serum IGF-I concentrations were determined by RIA (Incstar, Stillwater, USA) with a detection limit of 1.5 nmol/liter and an interassay CV less than 11%. Since 2005, serum IGF-I concentrations (nanomoles/liter) were measured using an immunometric technique on an Immulite 2500 system (Siemens Healthcare Diagnostics, Deerfield, USA). The intraassay variations at mean plasma levels of 8 and 75 nmol/liter were 5.0% and 7.5%, respectively. A Hitachi modular P800 autoanalyzer (Roche Diagnostics, Indianapolis, USA) was used to quantify fasting serum concentrations of glucose, total cholesterol, and triglycerides. Fasting serum concentrations of HDL-cholesterol were measured with a homogenous enzymatic assay (Hitachi 911; Roche).

### Statistical analysis

Standardized morbidity ratios were estimated based on observed and expected number of patients fulfilling the different metabolic syndrome criteria. Expected numbers were obtained by indirect standardization, in which population specific morbidity risks were applied to the number of persons at risk stratified for gender and age (<30, 30–40, 40–50, 50–60, 60–70, and >70 years old). The 95% confidence interval was calculated by a shortcut method based on the Poisson distribution [17].

VFD, radiotherapy, and (treated) hypopituitarism (defined as number of affected axes, 0–4) were tested for their influence on the different metabolic syndrome criteria using logistic regression. A minimally adjusted model included age and gender (model 1) or age, gender and BMI (model 2), to assess the total effect of a given variable (e.g. radiotherapy), irrespective of its causal pathway. A maximally adjusted model included age, gender, VFD, radiotherapy, and hypopituitarism, again without or with BMI (models 3 and 4, respectively), to assess the unmediated direct effect of the variable. In an additional analysis, hypopituitarism was replaced by ACTH deficiency, TSH deficiency, GHD, or rhGH replacement (in the GHD patients only). In another analysis, VFD was replaced by suprasellar tumor extension against the 3rd ventricle (Hardy-Wilson classification B or C). BMI was included to the minimal and maximal models separately because it can be considered both as closely related to waist circumference and therefore as part of the metabolic syndrome (arguing against correction), and as a confounding influence to the other parameters of the metabolic syndrome (arguing for correction).

## Results

### Clinical characteristics (Table 1)

**Table 1:** Characteristics of NFMA patients.

Characteristics	NFMA patients (N = 145)
Female	64 (44)
Age	63 (26–88)
VFD at presentation	125 (86)
Transsphenoidal surgery	145 (100)
Adjuvant radiotherapy	68 (47)
Years post-surgery	12 (1–49)
Body Mass Index^†^	27.6 ± 4.2
Hardy tumor size	
II. >10 mm without invasiveness	93 (64)
III. Localized invasive	32 (22)
IV. Diffuse invasive	20 (14)
Hardy tumor extension	
A. Suprasellar cistern	8 (6)
B. Recesses of 3rd ventricle	120 (83)
C. Whole anterior 3rd ventricle	14 (10)
D. Intracranial (intradural)	2 (1)
E. Into or beneath cavernous sinus	37 (26)
One or more pituitary deficiencies	134 (92)
ACTH deficiency	102 (70)
TSH deficiency	104 (71)
LF/FSH deficiency	94 (65)
GH deficiency	109 (75)
Uses rhGH^‡^	82 (75)
Stopped using rhGH^‡^	11 (10)
Antihypertensive use	59 (41)
Statin use	51 (35)
Fibrates or nicotinic acid use	1 (1)

Data represent number (percentage) or median (range). VFD: visual field defects. rhGH: recombinant human growth hormone. ^†^Data represent average ± standard deviation. ^‡^Percentage within growth hormone deficiency.

All 145 NFMA patients (64 women) were treated by transsphenoidal surgery for suprasellar extension and were in remission (considered as absence of or stable residual adenoma) for at least one year (median 12 yrs, range 1–49 yr). Median age at the time of current evaluation was 63 years (range 26–88 yrs). Preoperatively, 125 patients (86%) presented with VFD, which improved considerably after surgery in all patients, although small peripheral VFD persisted in 55% of patients. These patients did not differ in gender, age, BMI, radiotherapy use, or substitution therapy from the 45% that experienced full visual field recovery. Tumor size was larger than 10 mm in all cases, most often classified as non-invasive (64%) and extending suprasellarly (in the recesses of the third ventricle) in 83%. Adjuvant radiotherapy was required in 68 patients (47%) for recurrent or persistent disease. At the time of evaluation, 92% of patients had one or more pituitary deficiencies. Specifically, 75% had somatotrope deficiency, 70% corticotrope deficiency, and 71% thyrotrope deficiency. Gonadotrope deficiency was present in 69% of men and 59% of women (although 53% of women were postmenopausal at evaluation). All patients were adequately and stably treated for all pituitary deficiencies, except for GHD, which was untreated in 27 of 109 GHD patients (25%) because of contraindications or the patient’s preference. Antihypertensive medication was used by 41% of patients, statins by 35%, and fibrates or nicotinic acid by 1%.

Control data of 63,995 population samples controls (59% females) were used, with a median age of 45 years (range 18–80 yrs). In this population, 11% used antihypertensive medication, 5% statins, and 0% used fibrates or nicotonic acid.

### Prevalence of (components of) the metabolic syndrome (Figure 1)

**Figure 1:**
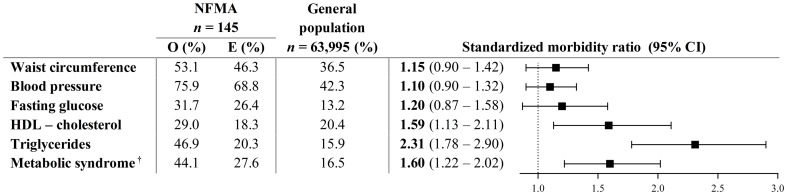
Comparison of metabolic syndrome prevalence, according to the NCEP-ATP III criteria, between NFMA patients and the general population. O, observed; E, expected. ^†^according to the NCEP-ATP III [10].

Of 145 NFMA patients, elevated waist circumference was observed in 53.1%, elevated blood pressure in 75.9%, elevated fasting glucose in 31.7%, reduced HDL-cholesterol in 29.0%, and elevated triglycerides in 46.9%. Subsequently, 44.1% of patients were classified as having the metabolic syndrome.

Compared to the general population, NFMA patients showed increased risk for reduced HDL-cholesterol (SMR 1.59, 95% CI 1.13–2.11), elevated triglycerides (SMR 2.31, 95% CI 1.78–2.90), and the metabolic syndrome (SMR 1.60, 95% CI 1.22–2.02). No increased risk for elevated waist circumference, blood pressure, or glucose levels was observed.

### Risk factors for metabolic parameters within the NFMA cohort (Table 2)

**Table 2:** Risk factors for the metabolic syndrome, according to the NCEP-ATPIII criteria, in NFMA patients using four logistic regression models.

Waist circumference				
	*OR_1_*	*OR_2_*	*OR_3_*	*OR_4_*
VFD	0.9 (0.4–2.4)	0.9 (0.3–3.2)	0.8 (0.3–2.2)	1.1 (0.3–3.7)
RT	1.2 (0.6–2.3)	1.2 (0.5–2.9)	1.0 (0.5–2.0)	1.3 (0.5–3.3)
Hypopit	1.6 (1.2–2.2)*	0.8 (0.5–1.1)	1.6 (0.2–2.2)	0.7 (0.5–1.1)

Fasting glucose				
	*OR_1_*	*OR_2_*	*OR_3_*	*OR_4_*
VFD	1.4 (0.5–4.3)	0.7 (0.2–2.1)	1.4 (0.5–4.1)	1.5 (0.5–4.5)
RT	1.0 (0.5–2.1)	1.0 (0.5–2.1)	1.0 (0.5–2.0)	1.0 (0.5–2.2)
Hypopit	1.2 (0.9–1.6)	0.9 (0.7–1.3)	1.2 (0.9–1.6)	0.9 (0.6–1.3)

Triglycerides				
	*OR_1_*	*OR_2_*	*OR_3_*	*OR_4_*
VFD	0.5 (0.2–1.4)	0.5 (0.2–1.4)	0.5 (0.2–1.3)	0.5 (0.2–1.4)
RT	0.9 (0.5–1.8)	0.9 (0.5–1.8)	0.9 (0.4–1.7)	0.9 (0.5–1.9)
Hypopit	1.2 (0.9–1.6)	1.0 (0.7–1.3)	1.2 (0.9–1.6)	1.0 (0.7–1.3)

Blood pressure				
	*OR_1_*	*OR_2_*	*OR_3_*	*OR_4_*
VFD	5.1 (1.6–15.7)*	5.4 (1.7–17.2)*	5.2 (1.7–16.3)*	6.5 (1.9–22.2)*
RT	0.7 (0.3–1.7)	0.7 (0.3–1.7)	0.7 (0.3–1.8)	0.8 (0.3–2.0)
Hypopit	1.0 (0.7–1.3)	0.7 (0.5–1.1)	1.0 (0.7–1.3)	0.7 (0.4–1.0)

HDL – cholesterol				
	*OR_1_*	*OR_2_*	*OR_3_*	*OR_4_*
VFD	0.9 (0.3–2.6)	0.9 (0.3–2.5)	0.9 (0.3–2.5)	0.9 (0.3–2.7)
RT	1.6 (0.8–3.2)	1.6 (0.8–3.4)	1.5 (0.7–3.1)	1.7 (0.8–3.6)
Hypopit	1.2 (0.9–1.6)	0.9 (0.6–1.3)	1.2 (0.9–1.6)	0.9 (0.6–1.3)

Metabolic syndrome^†^				
	*OR_1_*	*OR_2_*	*OR_3_*	*OR_4_*
VFD	2.0 (0.7–5.6)	2.4 (0.7–7.6)	1.8 (0.6–5.2)	2.6 (0.8–8.5)
RT	1.4 (0.7–2.7)	1.6 (0.7–3.5)	1.3 (0.7–2.6)	1.7 (0.8–3.9)
Hypopit	1.4 (1.1–1.9)*	0.8 (0.6–1.2)	1.4 (1.0–1.9)*	0.7 (0.5–1.1)

Data represent odds ratio (95% confidence interval). RT, radiotherapy; VFD, visual field defects at presentation; Hypopit, hypopituitarism (number of deficient pituitary axes). OR_1_: regression model 1 (age and gender). OR_2_: regression model 2 (age, gender, BMI). OR_3_: regression model 3 (age, gender, VFD, RT, hypopit). OR_4_: regression model 4 (age, gender, VFD, RT, Hypopit, BMI). ^†^according to the NCEP-ATP III [10]. *difference statistically significant (P ≤ 0.05).

Preoperative VFD were independently associated with elevated blood pressure in all models (OR_1_ 5.1, 95% CI 1.6–15.7; OR_2_ 5.4, 95% CI 1.7–17.2; OR_3_ 7.4, 95% CI 2.1–26.2; OR_4_ 7.6, 95% CI 2.0–28.2), but no associations were observed with any other parameter of the metabolic syndrome. Persistence of VFD, and years after surgery were of no significant influence to the metabolic parameters in any of the four models (data not shown), and neither was radiotherapy. When preoperative VFD were replaced with suprasellar tumor extension against the 3rd ventricle (Hardy-Wilson classification B or C), no association between suprasellar extension and any of the parameters was observed.

Hypopituitarism was associated with increased waist circumference when corrected only for age and gender (OR_1_ 1.6, 95% CI 1.2–2.2), and with the metabolic syndrome when corrected for age and gender (OR_1_ 1.4, 95% CI 1.1–1.9), or for age, gender, VFD, and radiotherapy (OR_3_ 1.4, 95% CI 1.0–1.9). Additional analysis of deficiency of separate axes, revealed that this association between hypopituitarism and metabolic parameters could mainly be attributed to the influence of GHD patients on increased waist circumference (OR_1_ 6.3, 95% CI 2.5–15.4), reduced HDL-cholesterol (OR_1_ 3.3, 95% CI 1.7–9.3), and the metabolic syndrome (OR_1_ 5.0, 95% CI 2.0–12.7), although only in the minimal model. Within the GHD patients, rhGH replacement was associated with a BMI-dependent increased odd for increased waist circumference (OR_1_ 3.3, 95% CI 1.3–8.3; OR_3_ 3.5, 95% CI 1.4–8.8), blood pressure (OR_3_ 3.4, 95% CI 1.0–11.9), and the metabolic syndrome (OR_1_ 2.5, 95% CI 1.0–6.4; OR_3_ 2.7, 95% CI 1.1–7.0), when compared to the GHD patients without rhGH replacement. Cortisol substitution was associated with a mildly decreased odd for decreased HDL-cholesterol in models 3 and 4 (OR_3_ 0.3, 95% CI 0.1–0.9; OR_4_ 0.3, 95% CI 0.1–0.9), and thyroid hormone substitution with increased waist circumference (OR_2_ 0.2, 95% CI 0.1–0.6; OR_4_ 0.2, 95% CI 0.0–0.6) and the metabolic syndrome (OR_2_ 0.4, 95% CI 0.1–1.0) in models that included correction for BMI.

## Discussion

This study demonstrates that patients from an unselected cohort of patients treated for NFMA have a 60% increased risk for the metabolic syndrome, mainly due to a 59% increased risk for reduced HDL-cholesterol and a 131% increased risk for elevated triglycerides, despite long-term remission.

To date, long-term data on metabolic outcome in exclusively NFMA cohorts are scarce, and limited to mortality studies. One study reported no increased cardiovascular or cerebrovascular mortality in a cohort of 192 adequately substituted NFMA patients [18], although no individual influences of substitution therapy were evaluated. Another study observed increased mortality by cardiovascular and respiratory disease in a cohort of 573 NFMA patients with variable adequacy of replacement therapy (SMR 1.70, 95% CI 1.34–2.15), although outcome was significantly better when compared with craniopharyngioma or other causes of hypopituitarism [19]. Lastly, a meta-analysis including 5412 female hypopituitary patients of heterogeneous origin, both of childhood and adult onset, but excluding previous GH or ACTH excess, and with variable adequacy of substitution therapy, found SMR’s of 1.21–3.80 [20]. Thus, these mortality studies suggest an adverse metabolic profile in NFMA patients. This study is the first to assess the prevalence of metabolic syndrome components in a homogeneous cohort of NFMA patients in which hormone replacement therapy was administered according to current guidelines, including rhGH replacement therapy in the majority of GHD patients.

NFMA patients often present with compression of the optic chiasm region, potentially also affecting the hypothalamic region. The hypothalamus is known to play a key role in the regulation of energy balance [21], [22]. In accordance, a syndrome known as hypothalamic obesity occurs after structural hypothalamic damage, e.g. in craniopharyngioma, after radiotherapy or trauma, and in genetic disorders such as Prader-Willi syndrome [5]. Several studies suggest an important role for the hypothalamic SCN in metabolism, and dysregulation was reported to be associated with metabolic syndrome in mice, in elderly and in cranial radiotherapy [23]–[26]. Furthermore, we previously reported that NFMA patients show alterations in sleep stages and sleep-wake rhythmicity [4], which are largely influenced by SCN function. Therefore, although sleep restriction per se is also directly linked to metabolic parameters [6], the metabolic alterations found in NFMA patients might be explained by mechanical destruction of hypothalamic nuclei, specifically the SCN, in addition to intrinsic imperfections of hormone replacement therapy for hypopituitarism [27] or untreated GH deficiency.

In the current study, preoperative VFD, indicating compression of the optic chiasm region, were independently associated with hypertension. Interestingly, postmortem neuroanatomical assessment of hypertensive patients revealed disturbed SCN structure [28], and re-entraining the SCN with melatonin in hypertensive patients allowed the physiological decrease in blood pressure at night to recur [29]. Melatonin also induces visceral fat loss and improvement of the metabolic syndrome in rats [30], although VFD were not associated with central obesity or the metabolic syndrome in the current study. It is of note that the duration of compression cannot be accurately evaluated, and that at present it is not possible to specifically measure hypothalamic function. In addition, it is not known whether VFD or only radiological evaluation are a requisite for hypothalamic compression. However, a recent post-mortem study in patients with suprasellar tumors leading to visual field defects reported reduced arginine-vasopressine immunoreactivity in the SCN, when compared to controls [31]. Notably, the association between suprasellar invasiveness (through the observation of preoperative VFD) and hypertension was not observed when a Hardy-Wilson classification of B (against recesses of 3rd ventricle) or C (whole anterior 3rd ventricle) was used to define hypothalamic compression. This might be explained by intra- and inter-observer variation, and the fact that the position of the compressing tumor relative to the SCN is not taken into account.

We further explored several factors with possible influence on (components of) the metabolic syndrome. Number of deficient pituitary axes (which might indicate larger tumor size) was associated with an increased odd for abdominal obesity and the metabolic syndrome, mainly due to the influence of GHD, although this association was dependent on BMI, VFD, radiotherapy and other pituitary deficiencies. Importantly, GHD did not influence the affected triglyceride component in our cohort. GHD can therefore potentially explain some, but not all of the metabolic risk in NFMA patients. In addition, we observed an increased odd for abdominal obesity, hypertension and the metabolic syndrome for rhGH replacement within GHD patients, although this might be confounded by treatment indication in this cross-sectional analysis. In previous studies, associations between untreated GHD and increased prevalence of dyslipidemia, hypertension, abdominal obesity and the metabolic syndrome were observed [32]–[34]. RhGH treatment improved dyslipidemia, hypertension and obesity in many [32], [35], but not all [34], [36] studies, and insulin sensitivity decreased in some studies [34]–[36]. The results in our cohort of NFMA patients were therefore partially in line with reports in GHD cohorts, although those studies are difficult to compare due to large heterogeneity.

In our cohort, glucocorticoid replacement was associated with a mildly decreased prevalence of lower HDL, but had no effect on the metabolic syndrome. Previous studies reported increased prevalence of the metabolic syndrome in mildly increased cortisol levels of overtreated NFMA patients [37]. All patients in our cohort were, however, stably and adequately substituted for ACTH deficiency at the lowest dose considered adequate on a daily basis (mean 20.7±4.2 mg). Radiotherapy and TSH deficiency did not influence components of the metabolic syndrome in our cohort.

Several limitations of the study need to be discussed. Patients with sufficient data were selected from a pre-existing database, inducing the possibility of a selection bias. For instance, metabolic parameters are in general more frequently evaluated in GHD patients, increasing their chance of being included. Correcting for hormonal deficiencies in the regression analysis minimalized this bias. Also, the cross-sectional design of this study did not allow us to study incidence and mortality rates, although we previously reported nonsignificantly increased overall mortality in 172 NFMA patients in our institution (SMR 1.24, 95% CI 0.85–1.74) [38]. Another limitation is the definition or classification of risk factors, e.g. for pituitary function, treatment modality and having a history of hypothalamic compression. Also, we did not correct for the number of years after radiotherapy or after start of rhGH, since patients were considered to be in a stable situation and the most dramatic effects of those treatments are observed in the short-term. However, their effect on the long-term might still influence the metabolic outcome to some extent, although not significantly in this study. Next, inherent to stratification is the chance of residual confounding. Within the strata, patients were statistically significantly older than the general population in age-groups 50–60 years (54.7±2.8 vs. 53.8±3.1, *P* = 0.05) and >70 years (76.3±4.7 vs. 73.7±2.7, *P*<0.001), although the clinical relevance of these differences can be considered small. Next, in this unselected cohort, most patients had tumors with supersellar tumor extension, as indicated by the prevalent preoperative VFD (86%) or Hardy’s classification B or C (93%). This uneven distribution might have limited the statistical power to assess differences in metabolic outcome caused by suprasellar extension. Lastly, we chose not to include statin use in the criteria for the metabolic syndrome, as directed in the official statement [10]. Consequently, since statins have a small beneficial effect on triglycerides and HDL, and statin use was higher in NFMA patients than controls (35% vs. 5.3%), it is likely that the results of this study underestimate the actual risk for the metabolic syndrome and represent a rather conservative estimation of the magnitude of the metabolic disadvantages that come with a history of NFMA.

In conclusion, patients treated for NFMA are at increased risk of having the metabolic syndrome, in particular dyslipidemia. This was only partly explained by hypopituitarism or hormone replacement therapy. Further study is required to determine which processes are involved in this patient group, e.g. by assessing alterations in circadian activity, food intake, or energy expenditure. The results of this study advocate stringent control and treatment of cardiovascular risk factors, like in patients with prevalent diseases at increased cardiovascular risk.

## References

[1] Daly AF, Rixhon M, Adam C, Dempegioti A, Tichomirowa MA, et al (2006) High prevalence of pituitary adenomas: a cross-sectional study in the province of Liege, Belgium. J Clin Endocrinol Metab 91: 4769–4775. 16968795 10.1210/jc.2006-1668

[2] Dekkers OM, de Keizer RJ, Roelfsema F, Vd Klaauw AA, Honkoop PJ, et al (2007) Progressive improvement of impaired visual acuity during the first year after transsphenoidal surgery for non-functioning pituitary macroadenoma. Pituitary 10: 61–65. 17318437 10.1007/s11102-007-0007-0 PMC1915635

[3] Park P, Chandler WF, Barkan AL, Orrego JJ, Cowan JA, et al (2004) The role of radiation therapy after surgical resection of nonfunctional pituitary macroadenomas. Neurosurgery 55: 100–106. 15214978 10.1227/01.neu.0000126885.71242.d7

[4] Biermasz NR, Joustra SD, Donga E, Pereira AM, van Duinen N, et al (2011) Patients previously treated for nonfunctioning pituitary macroadenomas have disturbed sleep characteristics, circadian movement rhythm, and subjective sleep quality. J Clin Endocrinol Metab 96: 1524–1532. 21367934 10.1210/jc.2010-2742

[5] Hochberg I, Hochberg Z (2010) Expanding the definition of hypothalamic obesity. Obes Rev 11: 709–721. 20233310 10.1111/j.1467-789X.2010.00727.x

[6] Knutson KL (2012) Does inadequate sleep play a role in vulnerability to obesity? Am J Hum Biol 24: 361–371. 22275135 10.1002/ajhb.22219 PMC3323702

[7] Hardy J (1979) The transsphenoidal surgical approach to the pituitary. Hosp Pract 14: 81–89. 10.1080/21548331.1979.11707562 571837

[8] Ghigo E, Aimaretti G, Corneli G (2008) Diagnosis of adult GH deficiency. Growth Horm IGF Res 18: 1–16. 17766155 10.1016/j.ghir.2007.07.004

[9] Stolk RP, Rosmalen JG, Postma DS, de Boer RA, Navis G, et al (2008) Universal risk factors for multifactorial diseases: LifeLines: a three-generation population-based study. Eur J Epidemiol 23: 67–74. 18075776 10.1007/s10654-007-9204-4

[10] Grundy SM, Cleeman JI, Daniels SR, Donato KA, Eckel RH, et al (2005) Diagnosis and management of the metabolic syndrome: an American Heart Association/National Heart, Lung, and Blood Institute Scientific Statement. Circulation 112: 2735–2752. 16157765 10.1161/CIRCULATIONAHA.105.169404

[11] Lorenzo C, Williams K, Hunt KJ, Haffner SM (2007) The National Cholesterol Education Program - Adult Treatment Panel III, International Diabetes Federation, and World Health Organization definitions of the metabolic syndrome as predictors of incident cardiovascular disease and diabetes. Diabetes Care 30: 8–13. 17192325 10.2337/dc06-1414

[12] de Simone G, Devereux RB, Chinali M, Best LG, Lee ET, et al (2007) Prognostic impact of metabolic syndrome by different definitions in a population with high prevalence of obesity and diabetes: the Strong Heart Study. Diabetes Care 30: 1851–1856. 17440172 10.2337/dc06-2152

[13] Ryan MC, Fenster Farin HM, Abbasi F, Reaven GM (2008) Comparison of waist circumference versus body mass index in diagnosing metabolic syndrome and identifying apparently healthy subjects at increased risk of cardiovascular disease. Am J Cardiol 102: 40–46. 18572033 10.1016/j.amjcard.2008.02.096

[14] Alberti KG, Zimmet P, Shaw J (2005) The metabolic syndrome—a new worldwide definition. Lancet 366: 1059–1062. 16182882 10.1016/S0140-6736(05)67402-8

[15] Law MR, Wald NJ, Rudnicka AR (2003) Quantifying effect of statins on low density lipoprotein cholesterol, ischaemic heart disease, and stroke: systematic review and meta-analysis. BMJ 326: 1423. 12829554 10.1136/bmj.326.7404.1423 PMC162260

[16] Stein EA, Lane M, Laskarzewski P (1998) Comparison of statins in hypertriglyceridemia. Am J Cardiol 81: 66B–69B. 10.1016/s0002-9149(98)00041-1 9526817

[17] Vandenbroucke JP (1982) A shortcut method for calculating the 95 percent confidence interval of the standardized mortality ratio. Am J Epidemiol 115: 304–305.

[18] Nielsen EH, Lindholm J, Laurberg P, Bjerre P, Christiansen JS, et al (2007) Nonfunctioning pituitary adenoma: incidence, causes of death and quality of life in relation to pituitary function. Pituitary 10: 67–73. 17356906 10.1007/s11102-007-0018-x

[19] Tomlinson JW, Holden N, Hills RK, Wheatley K, Clayton RN, et al (2001) Association between premature mortality and hypopituitarism. West Midlands Prospective Hypopituitary Study Group. Lancet 357: 425–431. 11273062 10.1016/s0140-6736(00)04006-x

[20] Nielsen EH, Lindholm J, Laurberg P (2007) Excess mortality in women with pituitary disease: a meta-analysis. Clin Endocrinol (Oxf) 67: 693–697. 17634076 10.1111/j.1365-2265.2007.02947.x

[21] Morton GJ, Cummings DE, Baskin DG, Barsh GS, Schwartz MW (2006) Central nervous system control of food intake and body weight. Nature 443: 289–295. 16988703 10.1038/nature05026

[22] King BM (2006) The rise, fall, and resurrection of the ventromedial hypothalamus in the regulation of feeding behavior and body weight. Physiol Behav 87: 221–244. 16412483 10.1016/j.physbeh.2005.10.007

[23] Kreier F, Yilmaz A, Kalsbeek A, Romijn JA, Sauerwein HP, et al (2003) Hypothesis: shifting the equilibrium from activity to food leads to autonomic unbalance and the metabolic syndrome. Diabetes 52: 2652–2656. 14578282 10.2337/diabetes.52.11.2652

[24] Coomans CP, van den Berg SA, Lucassen EA, Houben T, Pronk AC, et al (2013) The suprachiasmatic nucleus controls circadian energy metabolism and hepatic insulin sensitivity. Diabetes 62: 1102–1108. 23274903 10.2337/db12-0507 PMC3609590

[25] Hofman MA, Swaab DF (1994) Alterations in circadian rhythmicity of the vasopressin-producing neurons of the human suprachiasmatic nucleus (SCN) with aging. Brain Res 651: 134–142. 7922560 10.1016/0006-8993(94)90689-0

[26] Borgers AJ, Alkemade A, Venema HW, Fliers E, Bisschop PH (2012) A history of cranial radiotherapy is associated with a higher visceral to subcutaneous fat ratio in men with pituitary insufficiency. Eur J Endocrinol 166: 619–624. 22247015 10.1530/EJE-11-1023

[27] Romijn JA, Smit JW, Lamberts SW (2003) Intrinsic imperfections of endocrine replacement therapy. Eur J Endocrinol 149: 91–97. 12887284 10.1530/eje.0.1490091

[28] Goncharuk VD, van Heerikhuize J, Dai JP, Swaab DF, Buijs RM (2001) Neuropeptide changes in the suprachiasmatic nucleus in primary hypertension indicate functional impairment of the biological clock. J Comp Neurol 431: 320–330. 11170008 10.1002/1096-9861(20010312)431:3<320::aid-cne1073>3.0.co;2-2

[29] Scheer FA, Van Montfrans GA, Van Someren EJ, Mairuhu G, Buijs RM (2004) Daily nighttime melatonin reduces blood pressure in male patients with essential hypertension. Hypertension 43: 192–197. 14732734 10.1161/01.HYP.0000113293.15186.3b

[30] Wolden-Hanson T, Mitton DR, McCants RL, Yellon SM, Wilkinson CW, et al (2000) Daily melatonin administration to middle-aged male rats suppresses body weight, intraabdominal adiposity, and plasma leptin and insulin independent of food intake and total body fat. Endocrinology 141: 487–497. 10650927 10.1210/endo.141.2.7311

[31] Borgers AJ, Fliers E, Siljee JE, Swaab DF, Van Someren EJ, et al (2013) Arginine vasopressin immunoreactivity is decreased in the hypothalamic suprachiasmatic nucleus of subjects with suprasellar tumors. Brain Pathol 23: 440–444. 23278971 10.1111/bpa.12016 PMC8028940

[32] Verhelst J, Abs R (2009) Cardiovascular risk factors in hypopituitary GH-deficient adults. Eur J Endocrinol 161 Suppl 1S41–S49. 19684057 10.1530/EJE-09-0291

[33] van der Klaauw AA, Biermasz NR, Feskens EJ, Bos MB, Smit JW, et al (2007) The prevalence of the metabolic syndrome is increased in patients with GH deficiency, irrespective of long-term substitution with recombinant human GH. Eur J Endocrinol 156: 455–462. 17389460 10.1530/EJE-06-0699

[34] Attanasio AF, Mo D, Erfurth EM, Tan M, Ho KY, et al (2010) Prevalence of metabolic syndrome in adult hypopituitary growth hormone (GH)-deficient patients before and after GH replacement. J Clin Endocrinol Metab 95: 74–81. 19897679 10.1210/jc.2009-1326

[35] Maison P, Griffin S, Nicoue-Beglah M, Haddad N, Balkau B, et al (2004) Impact of growth hormone (GH) treatment on cardiovascular risk factors in GH-deficient adults: a Metaanalysis of Blinded, Randomized, Placebo-Controlled Trials. J Clin Endocrinol Metab 89: 2192–2199. 15126541 10.1210/jc.2003-030840

[36] Claessen KM, Appelman-Dijkstra NM, Adoptie DM, Roelfsema F, Smit JW, et al (2013) Metabolic profile in growth hormone-deficient (GHD) adults after long-term recombinant human growth hormone (rhGH) therapy. J Clin Endocrinol Metab 98: 352–361. 23162104 10.1210/jc.2012-2940

[37] Zueger T, Kirchner P, Herren C, Fischli S, Zwahlen M, et al (2012) Glucocorticoid replacement and mortality in patients with nonfunctioning pituitary adenoma. J Clin Endocrinol Metab 97: E1938–E1942. 22872686 10.1210/jc.2012-2432

[38] Dekkers OM, Biermasz NR, Pereira AM, Roelfsema F, van Aken MO, et al (2007) Mortality in patients treated for Cushing's disease is increased, compared with patients treated for nonfunctioning pituitary macroadenoma. J Clin Endocrinol Metab 92: 976–981. 17200171 10.1210/jc.2006-2112

